# Inequalities in diarrhoea, pneumonia and measles deaths: estimates for 21 sub-Saharan African countries

**DOI:** 10.2471/BLT.24.292198

**Published:** 2025-08-21

**Authors:** Stéphane Verguet, Dominick Villano, Boshen Jiao, Sarah Bolongaita, Isabelle Iversen, Ryoko Sato, Mieraf Taddesse Tolla, Solomon Tessema Memirie

**Affiliations:** a Department of Global Health and Population, Harvard T.H. Chan School of Public Health, 665 Huntington Avenue, Boston, MA 02115, United States of America.; b Health Economics & Financing Programme, Africa Centres for Disease Control and Prevention, Addis Ababa, Ethiopia.; c Addis Center for Ethics and Priority Setting, Addis Ababa University, Addis Ababa, Ethiopia.

## Abstract

**Objective:**

To develop new methods to analyse the distributions of diarrhoea, pneumonia and measles deaths in children younger than 5 years across wealth quintiles.

**Methods:**

We used Demographic and Health Surveys conducted since 2013 from 21 sub-Saharan African countries. We implemented multidimensional optimization techniques to estimate the joint impact of risk factors (that is, stunting, wasting, underweight, vitamin A deficiency and unsafe sanitation), immunization coverage and treatment utilization, on the distribution of deaths from diarrhoea, pneumonia and measles across wealth quintiles in each country. For each country, we created wealth-related gradients to show the risk of dying from either diarrhoea, pneumonia or measles.

**Findings:**

Across all countries and diseases, the risks of dying from diarrhoea, pneumonia and measles decrease with increasing household wealth: children in the wealthiest quintile are at the lowest risk (set to 1), except in a few rare instances. Yet, the magnitudes of these estimated risk gradients varied considerably across diseases and countries, from under 2 to above 10. Wealth-related risks of dying seemed to be unrelated to the countries’ levels of under-5 mortality.

**Conclusion:**

We estimate that inequalities in deaths from diarrhoea, pneumonia and measles are large in many countries of sub-Saharan Africa, with more deaths occurring among children in the poorest wealth quintiles compared with the richest. Our new and generalizable methods can help research on health disparities to explore new directions.

## Résumé

**Objectif:**

Mettre au point de nouvelles méthodes pour analyser la distribution des décès dus à la diarrhée, à la pneumonie et à la rougeole chez les enfants de moins de 5 ans dans tous les quintiles de richesse.

**Méthodes:**

Nous avons utilisé des enquêtes démographiques et de santé menées depuis 2013 dans 21 pays d’Afrique subsaharienne. Nous avons mis en œuvre des techniques d’optimisation multidimensionnelle pour estimer l’impact conjoint des facteurs de risque (c’est-à-dire le retard de croissance, l’émaciation, l’insuffisance pondérale, la carence en vitamine A et un assainissement inadéquat), de la couverture vaccinale et de l’utilisation de traitements, sur la répartition des décès dus à la diarrhée, à la pneumonie et à la rougeole dans tous les quintiles de richesse dans chaque pays. Pour chacun d’entre eux, nous avons élaboré des gradients liés à la richesse pour identifier le risque de décès dû à la diarrhée, à la pneumonie ou à la rougeole.

**Résultats:**

Dans tous les pays et pour toutes les maladies, les risques de décès dus à la diarrhée, à la pneumonie et à la rougeole diminuent proportionnellement à l’augmentation de la richesse des ménages: les enfants du quintile le plus riche courent le risque le plus faible (défini à 1), sauf dans quelques rares cas. Cependant, l’ampleur de ces gradients de risque estimés varie considérablement d’une maladie à l’autre et d’un pays à l’autre, allant de moins de 2 à plus de 10. Les risques de décès liés à la richesse ne semblaient pas liés aux niveaux de mortalité des enfants de moins de 5 ans dans les différents pays.

**Conclusion:**

Nous estimons que les inégalités dans les décès dus à la diarrhée, à la pneumonie et à la rougeole sont importantes dans de nombreux pays d’Afrique subsaharienne. Un nombre supérieur de décès est observé chez les enfants des quintiles les plus pauvres par rapport aux plus riches. Nos méthodes novatrices et généralisables permettent d’aider la recherche sur les disparités en matière de santé à explorer de nouvelles pistes.

## Resumen

**Objetivo:**

Desarrollar nuevos métodos para analizar la distribución de las muertes por diarrea, neumonía y sarampión en niños menores de 5 años según los quintiles de riqueza.

**Métodos:**

Se utilizaron los datos de las Encuestas demográficas y de salud realizadas desde 2013 en 21 países del África subsahariana. Se aplicaron técnicas de optimización multidimensional para estimar el impacto conjunto de los factores de riesgo (a saber, retraso del crecimiento, emaciación, bajo peso, deficiencia de vitamina A y saneamiento inseguro), la cobertura de inmunización y la utilización de tratamiento, en la distribución de las muertes por diarrea, neumonía y sarampión según los quintiles de riqueza en cada país. Para cada país, se elaboraron gradientes relacionados con la riqueza que muestran el riesgo de morir por diarrea, neumonía o sarampión.

**Resultados:**

En todos los países y enfermedades analizadas, los riesgos de morir por diarrea, neumonía o sarampión disminuyen a medida que aumenta la riqueza del hogar: los niños del quintil más rico presentan el menor riesgo (establecido en 1), salvo en contadas excepciones. Sin embargo, la magnitud de estos gradientes de riesgo estimados varió considerablemente entre enfermedades y países, desde valores inferiores a 2 hasta superiores a 10. Los riesgos de mortalidad relacionados con la riqueza no parecieron estar asociados con los niveles nacionales de mortalidad en menores de 5 años.

**Conclusión:**

Se estima que las desigualdades en las muertes por diarrea, neumonía y sarampión son marcadas en muchos países del África subsahariana, con una mayor proporción de muertes entre los niños pertenecientes a los quintiles de riqueza más bajos en comparación con los más altos. Los métodos nuevos y generalizables aquí propuestos pueden contribuir a que la investigación sobre disparidades en salud explore nuevas direcciones.

## ملخص

الغرض تطوير أساليب جديدة لتحليل توزيع وفيات الإسهال والالتهاب الرئوي والحصبة لدى الأطفال دون سن الخامسة عبر شرائح الثروة.

الطريقة استخدمنا المسوحات السكانية والصحية التي أُجريت منذ عام 2013 في 21 دولة من دول جنوب الصحراء الكبرى. وطبقنا تقنيات تحسين متعددة الأبعاد لتقدير الأثر المشترك لعوامل الخطر (أي التقزم، والهزال، ونقص الوزن، ونقص فيتامين أ، وعدم سلامة الصرف الصحي)، وتغطية التطعيم، والاستفادة من العلاج، على توزيع وفيات الإسهال والالتهاب الرئوي والحصبة عبر شرائح الثروة في كل دولة. ولكل دولة، أنشأنا تدرجات مرتبطة بالثروة لإظهار خطر الوفاة بسبب الإسهال أو الالتهاب الرئوي أو الحصبة.

النتائج في جميع الدول والأمراض، تنخفض مخاطر الوفاة بسبب الإسهال والالتهاب الرئوي والحصبة مع زيادة ثروة الأسرة: الأطفال في الخمس الأغنى هم الأقل عرضة للخطر (مُحدد على 1)، باستثناء بعض الحالات النادرة. ومع ذلك، فإن أحجام تدرجات المخاطر المقدرة هذه تختلف اختلافًا كبيرًا باختلاف الأمراض والدول، من أقل من 2 إلى أكثر من 10. ويبدو أن مخاطر الوفاة المرتبطة بالثروة لا علاقة لها بمستويات وفيات الأطفال دون سن الخامسة في الدول.

الاستنتاج نقدر أن التفاوتات في الوفيات الناجمة عن الإسهال والالتهاب الرئوي والحصبة كبيرة في العديد من دول جنوب الصحراء الكبرى، مع حدوث المزيد من الوفيات بين الأطفال في أفقر خمس ثروة مقارنة بالأطفال في أغنى خمس. يمكن لأساليبنا الجديدة والقابلة للتعميم أن تساعد في البحث في التفاوتات الصحية لاستكشاف اتجاهات جديدة.

## 摘要

**目的:**

旨在开发新方法,来分析财富五分位数中 5 岁以下儿童腹泻、肺炎和麻疹死亡人数的分布情况。

**方法:**

我们运用了 2013 年以来 21 个撒哈拉以南非洲国家开展的所有人口和保健调查相关数据。我们采用了多维优化技术来评估风险因素(即发育迟缓、消瘦、体重不足、维生素 A 缺乏症和不安全的卫生设施)、免疫覆盖率和治疗利用率对各个国家财富五分位数中腹泻、肺炎和麻疹所致死亡人数分布情况的共同影响。我们针对每个国家都绘制了财富相关梯度图,以直观地显示因腹泻、肺炎或麻疹导致的死亡风险。

**结果:**

在所有国家和疾病中,因腹泻、肺炎和麻疹导致的死亡风险随着家庭财富的增加而有所降低:除少数罕见情况之外,最富裕五分位数中儿童的死亡风险最低(设定为 1)。但是,这些估算的风险梯度数值在不同疾病和国家中存在显著差异,其数值大小从小于 2 到大于 10 不等。财富相关死亡风险似乎与各国 5 岁以下儿童死亡率水平毫不相关。

**结论:**

据我们估计,在撒哈拉以南非洲地区的许多国家中,腹泻、肺炎和麻疹所致死亡人数存在严重的不平等现象,与最富裕的财富五分位数相比,最贫困的财富五分位数中儿童的死亡人数明显更多。我们开发的可推广使用的新方法有助于开展健康差异研究,从而探索新方向。

## Резюме

**Цель:**

Разработать новые методы анализа распределения смертности от диареи, пневмонии и кори среди детей в возрасте до 5 лет по квинтилям благосостояния.

**Методы:**

Авторы использовали данные демографических и медико-санитарных обследований, которые проводились с 2013 года в 21 стране Африки в регионе южнее Сахары. Были использованы многомерные методики оптимизации для оценки совместного влияния факторов риска (задержка роста, истощение, недостаточная масса тела, дефицит витамина А и плохое санитарное состояние жилищ), охвата вакцинацией и использования лечения на распределение смертности от диареи, пневмонии и кори по квинтилям благосостояния в каждой из стран. Для каждой из стран были построены градиенты по благосостоянию, отражающие риск смертности от диареи, пневмонии или кори.

**Результаты:**

Совокупно по всем странам и заболеваниям оказалось, что риски смерти от диареи, пневмонии и кори снижаются с ростом благосостояния семьи: дети в наиболее обеспеченном квинтиле имели наиболее низкий риск (который был принят за 1), за исключением небольшого количества редких случаев. Однако величина этих оценочных градиентов риска существенно варьировалась в зависимости от болезни и страны – от менее 2 до более 10. Исследование не выявило корреляции между уровнем развития страны и связанным с благосостоянием риском смерти для детей в возрасте до 5 лет.

**Вывод:**

По оценкам авторов в показателях смертности от диареи, пневмонии и кори в странах Африки южнее Сахары наблюдаются значительные различия, при этом регистрируется большее число смертей среди детей, относящихся к наиболее бедным квинтилям благосостояния, по сравнению с детьми из наиболее обеспеченных квинтилей. Эти новые генерализующие методы позволяют открыть новые направления для исследования проблем неравенства в сфере здравоохранения.

## Introduction

Diarrhoea, pneumonia and measles are leading causes of death in children younger than five years in low- and middle-income countries.^1^ Although child mortality has fallen worldwide over the last decades, this decline hides regional, subnational and wealth disparities.^2^^–^^4^ For example, in 2021, about one child in 14 would still die before their fifth birthday in sub-Saharan Africa.^2^ Within sub-Saharan Africa, under-five mortality rates vary considerable between countries, from 33 per 1000 live births in South Africa to 115 per 1000 live births in Niger.^2^ Poor communities, with low education levels, and/or located in rural areas often share a disproportionate burden of child mortality.^2^^,^^3^

Over the past 30 years, immunization – a very cost-effective public health intervention – has played an important role in the global decline of under-five mortality.^5^^,^^6^ However, there are still inequalities in immunization coverage. For example, while the estimated global proportion of one-year-old children who received a first dose of measles-containing vaccine increased from 72% to 82% between 2000 and 2021, the corresponding changes were 37% to 54% in Ethiopia, 46% to 55% in the Democratic Republic of the Congo and 33% to 59% in Nigeria.^7^ Within countries, subnational and socioeconomic disparities also exist.^3^ Large wealth-related gaps in coverage for measles, rotavirus and pneumococcal conjugate vaccines, coupled with underlying risk factors like stunting, wasting or underweight, and the access to and use of effective treatment for diarrhoea and pneumonia, could explain the inequalities in diarrhoea, pneumonia and measles deaths.^8^^,^^9^

To address these mortality disparities, evidence is needed to determine the most appropriate health policies for implementation. However, empirical reports and estimations of major under-five mortality causes, disaggregated by socioeconomic status, are lacking for sub-Saharan Africa. Most published studies use large household surveys, such as the Demographic and Health Surveys (DHS), to describe the distribution of disease incidence, prevalence or intervention coverage across wealth quintiles.^4^^,^^10^^–^^12^ Few studies have systematically attempted to estimate disease-specific mortality distributions by wealth quintile within countries;^8^^,^^9^ and the Global Burden of Disease study examines sociodemographic differences at a global level across countries.^1^

Therefore, we aimed to develop new methods to analyse the distributions of diarrhoea, pneumonia and measles deaths across wealth quintiles. We illustrate the methods by examining 21 countries in sub-Saharan Africa, using data from DHS.^3^ Specifically, we design and implement multidimensional optimization techniques to estimate the joint impact of risk factors (such as stunting, wasting and underweight), immunization coverage and treatment utilization on the distribution of deaths. We tested the hypothesis that disease-specific mortality is disproportionally concentrated among the poorest, as they often have more risk factors and lower immunization and treatment coverages.^3^

## Methods

### Data sources

We used data from recent DHS conducted in 21 countries in sub-Saharan Africa (Table 1). These nationally representative surveys included information on women aged 15–49 years and their children, including children’s nutritional status; water, sanitation and hygiene conditions; coverage of certain health interventions, such as immunizations and treatments; and mother’s sociodemographic characteristics.^3^^,^^13^ We deemed the DHS as the best available data sources for the purpose of our methods development, because of the data sets’ consistent reporting quality and comparability over time and across countries, and due to the availability of health indicators across wealth quintiles for children younger than five years.

**Table 1:** List of sub-Saharan African countries and year(s) of Demographic and Health Surveys used to estimate risk gradients for deaths from diarrhoea, pneumonia and measles

Country	DHS survey year(s)	Under-5 mortality (at survey year), deaths per 1000 live births
Angola	2015–2016	84
Burundi	2016–2017	62
Cameroon	2018	80
Chad	2014–2015	129
Democratic Republic of the Congo	2013–2014	99
Gambia	2019–2020	49
Ghana	2014	58
Guinea	2018	105
Kenya	2014	48
Lesotho	2014	83
Malawi	2015–2016	53
Mali	2018	106
Namibia	2013	47
Rwanda	2019–2020	41
Senegal	2019	42
Sierra Leone	2019	112
South Africa	2016	36
Togo	2013–2014	78
United Republic of Tanzania	2015–2016	52
Zambia	2018	63
Zimbabwe	2015	61

DHS: Demographic and Health Survey.

### Risk and prognostic factors

We restricted our examination of under-five mortality to three major causes of death: diarrhoea, pneumonia and measles. We selected these three causes as: (i) they are among the leading causes of mortality among children younger than five years in sub-Saharan Africa;^1^ (ii) there are well-documented risk and prognostic factors for these three diseases, that is individual characteristics that could determine the probability of acquiring and dying from each disease;^8^^,^^14^^–^^20^ and (iii) effective vaccines exist that could partially prevent rotavirus-caused diarrhoea, pneumococcal pneumonia and *Haemophilus influenzae* type b (Hib) pneumonia and measles.^21^^–^^25^

Building on previous research,^8^^,^^9^ we selected risk factors for mortality and morbidity and assigned corresponding relative risks for each disease (Table 2). We investigated six outcomes for each country: diarrhoea cases and deaths; measles cases and deaths; and pneumonia cases and deaths. The risk factors assigned to each of these six outcomes included childhood stunting, wasting and underweight; unsafe sanitation; and vitamin A deficiency. We considered medical importance, data availability and computational implementation feasibility for each risk factor, and determined that including up to four risks per outcome was sufficient to achieve precision and insight saturation.

**Table 2:** Selected risk and prognostic factors, for morbidity and mortality, respectively, for diarrhoea, pneumonia and measles

Disease, risk factors	Prognostic factors
**Diarrhoea**	
- Stunting;^14^^–^^17^ - Underweight;^14^^–^^16^^,^^18^ - Unsafe sanitation^15^^,^^16^^,^^18^ - Wasting^14^^–^^16^^,^^18^	- Stunting;^14^^–^^16^^,^^18^ - Underweight;^14^^–^^16^^,^^18^ - Wasting^14^^–^^16^^,^^18^
**Pneumonia**	
- Underweight^14^^,^^15^^,^^20^ - Vitamin A deficiency^14^^,^^15^^,^^17^^,^^20^ - Wasting^14^^,^^15^^,^^20^	- Stunting^14^^,^^15^^,^^20^ - Underweight^14^^,^^15^^,^^17^ - Vitamin A deficiency^14^^,^^15^^,^^17^^,^^20^ - Wasting^14^^,^^15^^,^^20^
**Measles**	
- Stunting^14^^,^^15^ - Underweight;^14^^,^^15^ - Vitamin A deficiency^14^^,^^15^^,^^19^ - Wasting^14^^,^^15^	- Stunting^14^^,^^15^ - Underweight^14^^,^^15^ - Vitamin A deficiency^14^^,^^15^^,^^19^ - Wasting^14^^,^^15^

Note: We adapted risk and prognostic factors from Chang et al., 2018^8^ and Bolongaita et al., 2022.^9^

### Risk profiles and optimization

After selecting risk factors for each of the six outcomes (Table 2), we created a synthetic population for the analysis, containing only children for whom all data for the corresponding risk factors were available from the DHS. While this synthetic population might slightly affect the national representativeness of the final sample, a complete risk factor data set was necessary for implementing our optimization methods.

We divided this synthetic population into subpopulations according to each individual’s risk profile. As an example, consider a population and disease for which the two relevant risk factors are stunting and wasting. Each risk factor has three possible levels: not stunted, stunted and severely stunted; and similarly for wasting, resulting in nine possible risk profiles based on the combination of the two risk factors. As the number of subpopulations increases multiplicatively with the number of risk factors, a large number of possible risk profiles might be computationally challenging during optimization, which was another reason for only including four risk factors.

Subsequently, we assigned unknown probabilities of either morbidity or mortality to each of the subpopulations created. The size of each subpopulation and the relative risks assigned to each risk factor then yielded a system of linear equations where the unknown variables were the probabilities of either morbidity or mortality, associated to either diarrhoea, measles or pneumonia. In general, there would be no exact probability solutions to this system of linear equations (online repository).^26^ Therefore, we aimed to minimize errors by identifying probabilities that achieved the lowest possible error.

In searching for these non-unique error-minimizing probabilities, we had to impose the following constraint: if the first subpopulation was equally or more at risk in all dimensions than the second subpopulation, we imposed that the probability assigned to the first subpopulation was greater than or equal to the probability assigned to the second subpopulation.

Subsequently, with our system of linear equations supplemented by the constraint we searched for error-minimizing probabilities by assigning random admissible combinations of unknown probabilities in the system of equations and iteratively seeking combinations that best fitted (that is, optimized) the system. This step was repeated 1000 times, thus yielding 1000 distinct probability combinations with means, medians and uncertainty intervals (UI) for each combination.

All details on our methods are available in the online repository.^26^

### Coverages

By assigning probabilities to each subpopulation in each DHS, we could derive a probability distribution for the number of disease cases or deaths within the whole population of each DHS. First, we only considered the underlying risk factors. Subsequently, we altered this distribution by further applying immunization coverage to each wealth quintile (as reported in the DHS). We also further applied treatment coverage (proxied by care-seeking, as reported in the DHS).

For rotavirus-caused diarrhoea, we used immunization coverage and efficacy for rotavirus vaccine. For measles, the approach was similar. For pneumonia, we examined both pneumococcal conjugate and Hib vaccines. We scaled each vaccine efficacy based on the proportions of diarrhoea or pneumonia cases attributable to the causative agent targeted by the vaccine (Table 3). For countries where health authorities had not included the relevant vaccines in the national immunization programme at the time the DHS was conducted, we set the corresponding immunization coverage rates to zero. We used care-seeking percentages reported in DHS as a proxy for treatment coverage for diarrhoea and pneumonia. As care-seeking behaviours for pneumonia are not reported in DHS, we used acute respiratory infections as a proxy; if not available, we used fever. For measles, we computed treatment coverage as the average of diarrhoea and acute respiratory infections care-seeking percentages (further details available in online repository).^26^ We assumed that effective treatment coverage could be approximated by care-seeking percentages for diarrhoea, acute respiratory infections and fever, despite potential recall and social biases. In the absence of other readily available data inputs on effective coverage that are consistent across wealth quintiles in all countries, these were the best data inputs we could use.

**Table 3:** Vaccine and treatment efficacies for rotavirus diarrhoea, pneumonia (pneumococcal and Hib) and measles used in the risk gradient estimation model

Disease	Vaccine	Vaccine efficacy (95% UI)	Treatment efficacy (95% UI)
Rotavirus diarrhoea	Rotavirus vaccine	0.50 (0.11–0.72)^21^	0.93 (0.83–0.98)^22^
Pneumonia			0.70 (0.52–0.82)^24^
Pneumococcal (33.0% of pneumonia)^21^	Pneumococcal conjugate vaccine	0.58 (0.29–0.75)^24^	
Hib (21.6% of pneumonia)^21^	Penta-3 vaccine	0.93 (0.83–0.97)^25^	
Measles	Measles-containing vaccine	0.85 (0.83–0.87)^23^	0.62 (0.52–0.82)^23^

Hib: *Haemophilus influenzae* type b; Penta-3: 3^rd^ dose of diphtheria, pertussis, tetanus, hepatitis B and *H. influenzae* type b; UI: uncertainty interval.

### Linking morbidity and mortality

When considering the estimation of deaths, we linked deaths to cases using three approaches, each of them being a distinct and simple model for capturing case fatality ratios. First, we computed number of deaths independently of number of cases using the methods described above. Second, we computed number of deaths using the risk factors for cases, and then applying both immunization and treatment coverage rates. Third, we used the number of cases directly from the error-minimizing probabilities exercise (optimization) and extrapolated them to estimate the number of deaths, using under-five mortality differential estimates disaggregated by wealth quintile as reported in the DHS. In our main analysis, we reported on the average of the first and third approaches.

### Reporting

For each country, we estimated and reported on the probability distributions corresponding to diarrhoea deaths, pneumonia deaths and measles deaths. By disaggregating by wealth quintile, we obtained five subdistributions per outcome and country, whose mean values we computed and normalized. This approach created a risk gradient for death from either diarrhoea, pneumonia or measles. We assigned a reference value of 1 to the quintile with the lowest risk of death (in almost all cases, the wealthiest quintile, V). We then compared the risk of death in this quintile with the other four quintiles (in most cases quintiles I–IV).

We conducted all simulations using the R software (R Foundation, Vienna, Austria).^27^

## Results

Of the included countries, Chad (DHS 2014–2015) and Sierra Leone (DHS 2019) had the highest levels of under-five mortality, with rates of 129 deaths and 112 deaths per 1000 live births, respectively. Whereas South Africa (DHS 2016) and Rwanda (DHS 2019–2020) had the lowest levels, with rates of 36 deaths and 41 deaths per 1000 live births, respectively (Table 1).

**Fig. 1:**
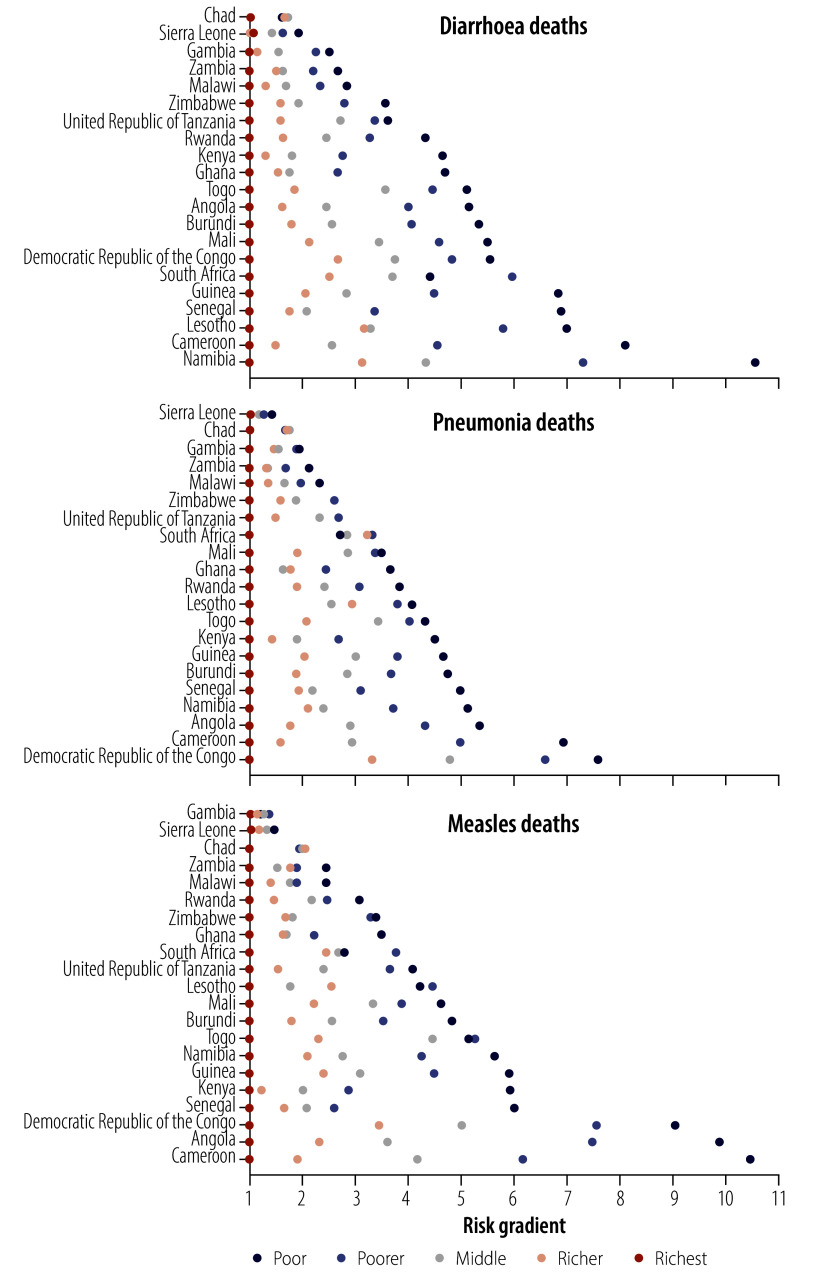
Estimated risk gradients for deaths from diarrhoea, pneumonia or measles in children younger than 5 years, by household wealth, 21 sub-Saharan African countries

The risk gradients for diarrhoea, pneumonia and measles, by wealth quintile, for each of the 21 countries are shown in Fig. 1. Consistent with our initial hypothesis, across all countries and diseases, the wealthiest quintile would be the least at risk of death, except for diarrhoea deaths in Sierra Leone (DHS 2019). Furthermore, almost all estimated risk gradients are monotonic, that is, as wealth decreases, the risk of dying increases. However, the magnitudes of these estimated risk gradients varied considerably across diseases and countries. For example, all the maximum risk estimates from Chad were under 2, despite having the highest under-five mortality rate. All the maximum risk estimates from Cameroon (DHS 2018) were above 6, the greatest of which was above 10, and the country had a relatively high under-five mortality rate (80 per 1000 live births; Table 1). Diarrhoea deaths in the poorest quintile in Namibia (DHS 2013) had the highest maximum risk estimate (around 11), despite that the under-five mortality rate was relatively low in the country (with 47 per 1000 live births; Table 1).

## Discussion

In this paper, we report on a quantitative method for estimating disease-related mortality rates across wealth quintiles in 21 sub-Saharan African countries with a high child mortality due to major infectious diseases, such as diarrhoea, measles and pneumonia. The methodological approach incorporated a multidimensional risk factor profile of a given population along with coverage rates of key health services, such as immunization and treatment. To populate the model, we used large comparable nationally representative household surveys.

We show that the risk gradients produced by our method can identify the extent to which underlying risk factors (in this study: stunting, wasting, underweight, vitamin A deficiency and/or unsafe sanitation), immunization or treatment coverages contribute to the disparities in diarrhoea, pneumonia and measles deaths. Greater socioeconomic disparities resulted in wider estimated risk gradients. Therefore, the results suggest that both social and health-care determinants can be linked to the disparities seen in deaths caused by major infectious diseases in sub-Saharan Africa.

The findings from this analysis are consistent with other published studies that reported on empirical observations of socioeconomic gradients in childhood diseases. Several studies, often leveraging nationally representative surveys such as the DHS, have either pointed to or estimated the greater risks of morbidity and mortality among the poorest households for diseases such as diarrhoea, pneumonia, acute respiratory infections and malaria in sub-Saharan Africa.^28^^–^^31^

The optimization methods employed in this paper are generalizable and could be applied to a wide variety of outcomes and disease areas, including to the distribution of mortality and causes across other stratifications than wealth, for example, education level, geographical units like regions, states and provinces.^9^ Yet, the analysis has several important limitations. First, the risk gradients estimates spanned many sub-Saharan African countries and survey data corresponded to different years (ranging from 2013 to 2020). Therefore, comparisons of gradients between countries should be made with caution, as the overall circumstances in under-five mortality and burdens of diarrhoea, pneumonia and measles could vary considerably across countries. Furthermore, there are inherent differences in the construction and comparability of wealth quintiles across countries.

Second, the intervention coverage indicators (that is, immunization coverage and care-seeking behaviours for diarrhoea and pneumonia treatment) were not readily available in all DHS, nor had consistent reporting quality, making comparison across countries and over time difficult. Furthermore, care-seeking percentages only represented an approximate proxy for effective treatment coverage. Likewise, we included only the first dose of measles-containing vaccine because many of the DHS did not routinely report on the second dose, either because of lack of implementation or due to low coverage. While two doses of measles-containing vaccine are recommended for controlling and preventing measles, the efficacy of the first dose remained sufficiently high (estimated at 85%)^23^ for the modelling purposes of our analysis.

Third, due to lack of data, the computational model we developed was static and did not account for any possible disease transmission dynamics or herd effects, which could be particularly important for infectious diseases with a large reproductive number, such as measles.^32^

Fourth, the creation of multidimensional risk factor profiles relied on the completeness of input data from the DHS. Since we made no assumptions on the background distribution of these profiles, we only included individuals with complete data profiles, as provided by the DHS, in the analysis. In some instances, this approach substantially reduced the number of individual data points available for analysis, resulting in limited national representativity and smaller sample size of the retained input data. 

Fifth, several technical limitations exist. The most notable is the maximum number of risk factors that we could consider for each disease. In our experience, computations involving more than four risk factors were often computationally intractable. Yet, even a substantial improvement in computational speed would probably not resolve the issue, as including additional risk factors would also require more demands on the requested number of data inputs. Another important technical limitation was the difficulty of uniformly sampling from admissible minimizing probability solutions during the constrained optimization procedure. We therefore used a straightforward rearrangement scheme that was easy to implement but was not uniform. Therefore, the possibility exists that certain sections of admissible minimizing probability solutions were not appropriately represented in our final results.

To conclude, we present new and generalizable methods for computing inequalities in health outcomes related to under-five mortality caused by major infectious diseases in low- and middle-income countries. The most novel aspect of this method is the use of constrained optimization procedures to compute multidimensional risk factor profiles, which we used for estimating disparities in risk gradients for a variety of countries in sub-Saharan Africa. We hope that our methods can point to new directions for research concerning health disparities, especially those that consider higher dimensional and joint probability distributions of social and care determinants of health.
